# Metastatic extra-axial medulloblastoma involving the trigeminal nerve: a rare prognostic entity with a comprehensive literature review

**DOI:** 10.3389/fonc.2025.1573781

**Published:** 2026-01-19

**Authors:** Federica D’Antonio, Andrea Carai, Giada del Baldo, Sabrina Rossi, Giovanna Stefania Colafati, Eleonora Piccirilli, Sabina Barresi, Isabella Giovannoni, Veronica Capelli, Selene Cipri, Rita Alaggio, Antonella Cacchione, Angela Mastronuzzi

**Affiliations:** 1 Department of Haematology-Oncology and Cell and Gene Therapy, Bambino Gesù Children Hospital, Istituto di Ricovero e Cura a Carattere Scientifico (IRCCS), Rome, Italy; 2 Onco-Hematology, Cell Therapy, Gene Therapies and Hemopoietic Transplant Unit, Bambino Gesù Children's Hospital, IRCCS, Rome, Italy; 3 Neurosurgery Unit, Department of Neuroscience and Neurorehabilitation, Bambino Gesù Children’s Hospital, Istituto di Ricovero e Cura a Carattere Scientifico (IRCCS), Rome, Italy; 4 Pathology Unit, Bambino Gesù Children’s Hospital, Istituto di Ricovero e Cura a Carattere Scientifico (IRCCS), Rome, Italy; 5 Oncological Neuroradiology Unit, Imaging Department, Bambino Gesù Children’s Hospital, Rome, Italy; 6 Pathology Unit, Department of Laboratories, Bambino Gesù Children’s Hospital, Istituto di Ricovero e Cura a Carattere Scientifico (IRCCS), Rome, Italy; 7 Department of Life Sciences and Public Health, Università Cattolica del Sacro Cuore, Rome, Italy

**Keywords:** extra-axial medulloblastoma, extra-neural metastases, genetic variants, molecular profiling, pediatric central nervous system tumors, rare entity, trigeminal nerve

## Abstract

Medulloblastomas (MB) are the most common malignant central nervous system tumors in children. They usually develop in the cerebellar vermis or the fourth ventricle, while in adults they typically originate from the paramedian region or the lateral cerebellar hemispheres. It’s rare for MBs to originate outside the brain and spinal cord (extra-axial), such as in the skull, meninges, and nerves. Metastases of MB typically occur within the central nervous system, with metastases outside the nervous system (extra-neural) being uncommon at the time of diagnosis (1-2%), but can increase to 5-10% during advanced stages. Around 5-6% of MBs are associated with inherited cancer predisposition syndromes, with common genetic variants including PTCH1, SUFU, TP53, and SMO. This report describes the first pediatric patient harboring a CHEK2 germline variant of uncertain significance and developing a EA- MB localized at the trigeminal nerve and subsequent CNS and EN metastases.

## 1 Introduction

Medulloblastomas (MB) are the most common malignant brain tumor in children accounting for 25% of pediatric central nervous system (CNS) tumors, with a median age at diagnosis of 6 years (1).

MBs are embryonal tumors derived from precursor cell populations and signaling pathways in the cerebellum, giving rise to various subtypes of MB. These tumors are postulated to originate from primitive, pluripotent neuroepithelial stem cells, differentiating along both glial and neuronal pathways *in situ* (2–4). Extra-axial medulloblastoma (EA-MB) is defined as a medulloblastoma arising outside the cerebellar parenchyma, such as from the cerebellopontine angle, calvarium, meninges, or cranial nerves. This presentation is extremely rare, accounting for less than 2% of all medulloblastoma cases, since most tumors are intra-axial and located in the midline cerebellar vermis or hemispheres. Extra- axial cases are typically described as isolated case reports or small series, and their diagnosis can be challenging due to overlap with more common extra-axial tumors such as meningioma or schwannoma. Clinical manifestations depend on the site of origin and may include cranial nerve deficits or atypical posterior fossa symptoms. Molecular subtyping (WNT, SHH, Group 3, Group 4) is relevant for prognosis and management; however, data on extra-axial cases remain limited (5, 6). Although medulloblastomas usually metastasize within the cerebrospinal fluid, evidence supports the possibility of hematogenous spread. Extra-neural metastasis (ENM) involves systemic dissemination, most often to bone, bone marrow, lymph nodes, lungs, and liver. ENM is rare at the time of initial diagnosis (1–2%), but its incidence increases to 5–10% in advanced or recurrent disease (7–9). Although most cases are sporadic, inherited cancer predisposition syndromes (CPS) have been associated with 5–6% of MB diagnoses (3, 8). The most common germline pathogenic variants associated with medulloblastoma include alterations in genes of the WNT signaling pathway, PTCH1 and SUFU (both associated with Gorlin syndrome, a condition predisposing to medulloblastoma), TP53 (associated with Li-Fraumeni syndrome), and SMO (associated with Curry-Jones syndrome). Additionally, familial adenomatous polyposis (FAP) has been rarely linked to the WNT subgroup of medulloblastomas.

Few cases in the literature report MBs associated with variants of *CHEK2* (checkpoint kinase 2), an onco-suppressor gene encoding the checkpoint kinase involved in the DNA damage response (10–13). This report aims to describe the first pediatric case of EA- MB involving the trigeminal nerve with CNS and extra-neural metastases. Interestingly, the patient harboured a germline variant of CHEK2 of uncertain significance.

## 2 Case report

A 6-year-old Caucasian male was admitted to the emergency department presenting with headache and vomiting associated with strabismus and right eye visual acuity loss.

The patient had no significant previous medical history and was the second child of healthy nonconsanguineous parents. Family history revealed that his maternal grandmother had died of breast cancer at the age of 50 and his maternal grandfather died at age 56 from an unspecified cancer.

**Figure 1:**
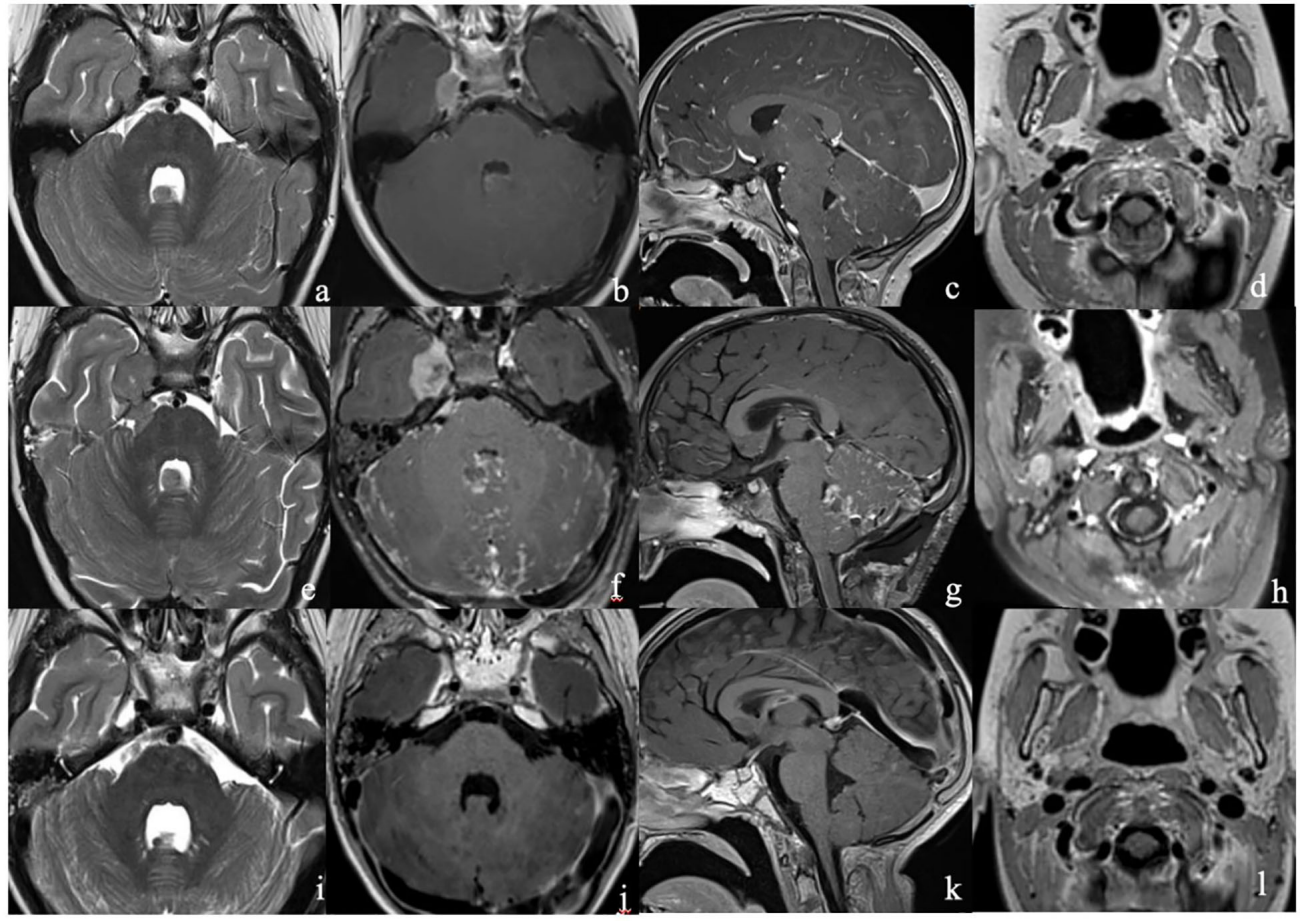
**(a–d)** MR at clinical presentation (upper row) demonstrated a conspicuous expansile lesion in the right Meckel’s cave extending into the cavernous sinus [white arrow, **(a, b)**], as well as a smaller pseudo- nodular lesion abutting the cerebellar vermis into the IV ventricle [white arrowhead, **(a, b)**]; a linear enhancement among the cerebellar folia **(c)** and the cord surface (not shown), consistent with leptomeningeal dissemination, could also be appreciated. The right parotid gland appears unremarkable; **(e–h)**] MR after 2 weeks from the initial MR (middle row) demonstrates an increase in size of the lesions in the right Meckel’s cave [white arrow, **(a, b)**] and cerebellar vermis [white arrowhead, **(a, b)**], and also an increase of leptomeningeal enhancement among the cerebellar folia **(g)**. A nodular lesion also appeared in the right parotid gland (yellow circle); **(i–l)**]MR after radiation therapy (lower row) showed a marked reduction of all the lesions.

**Figure 2:**
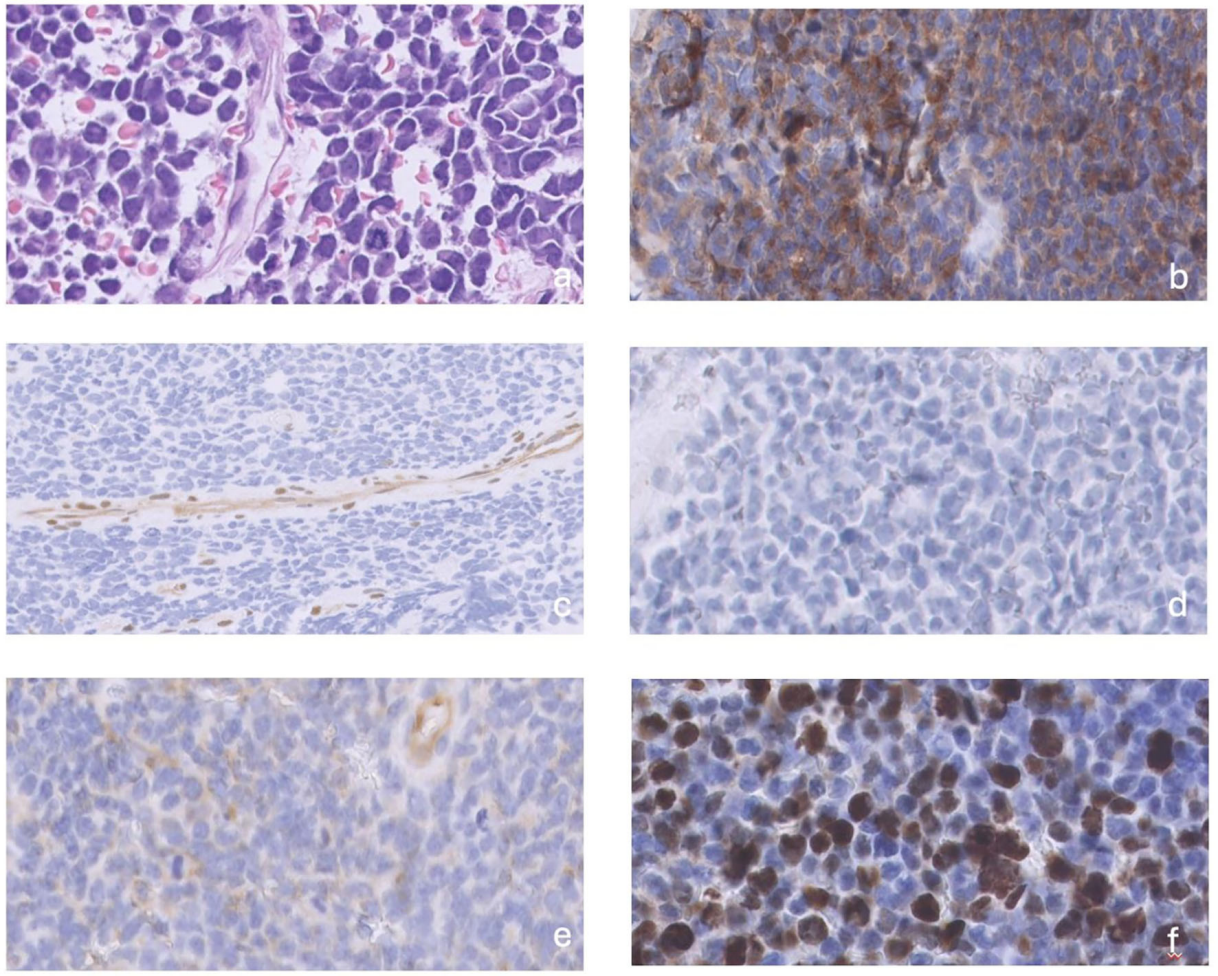
**(a)** primary tumors consisted of a solid growth pattern with small cells, high nucleus-to- cytoplasm ratio, hyperchromatic and slightly pleomorphic nuclei, sometimes arranged at the periphery of the cell, and inconspicuous nucleoli, **(b)** Synaptophysin was positive, **(c)** YAP1 was negative, **(d)** GAB1 was negative, **(e)** Beta-catenin expression was restricted to the cytoplasm, **(f)** mitoses were brisk (arrow).

**Figure 3:**
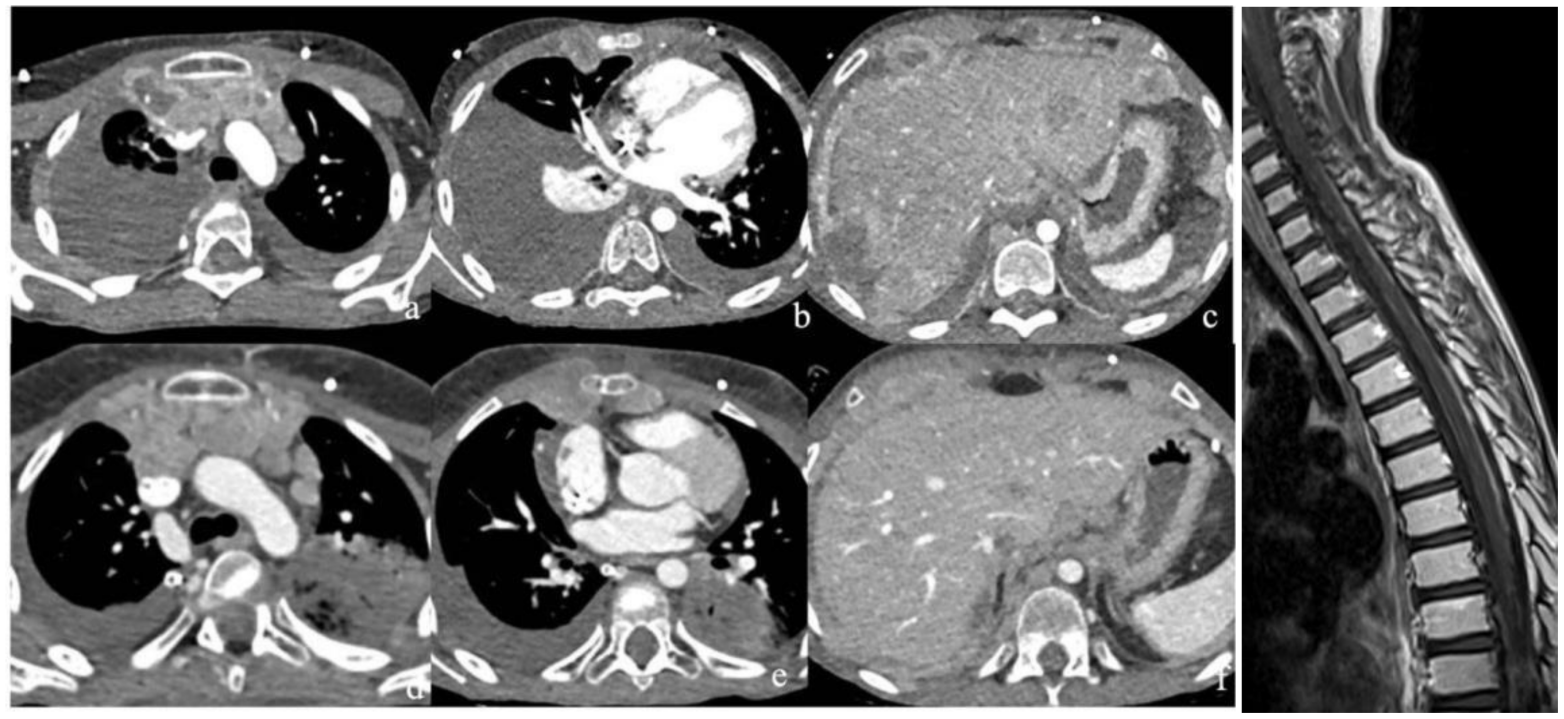
**(a–c)** CT obtained soon after the clinical deterioration of the patient (upper row) documented disease progression in the chest and abdomen due to the appearance of multiple lymphadenopathies and pleural and peritoneal nodules with corresponding effusion. There was concurrent CNS progression at MR (g); **(d–f)** Follow-up CT one month later (lower row) showed further disease progression.

**Figure 4:**
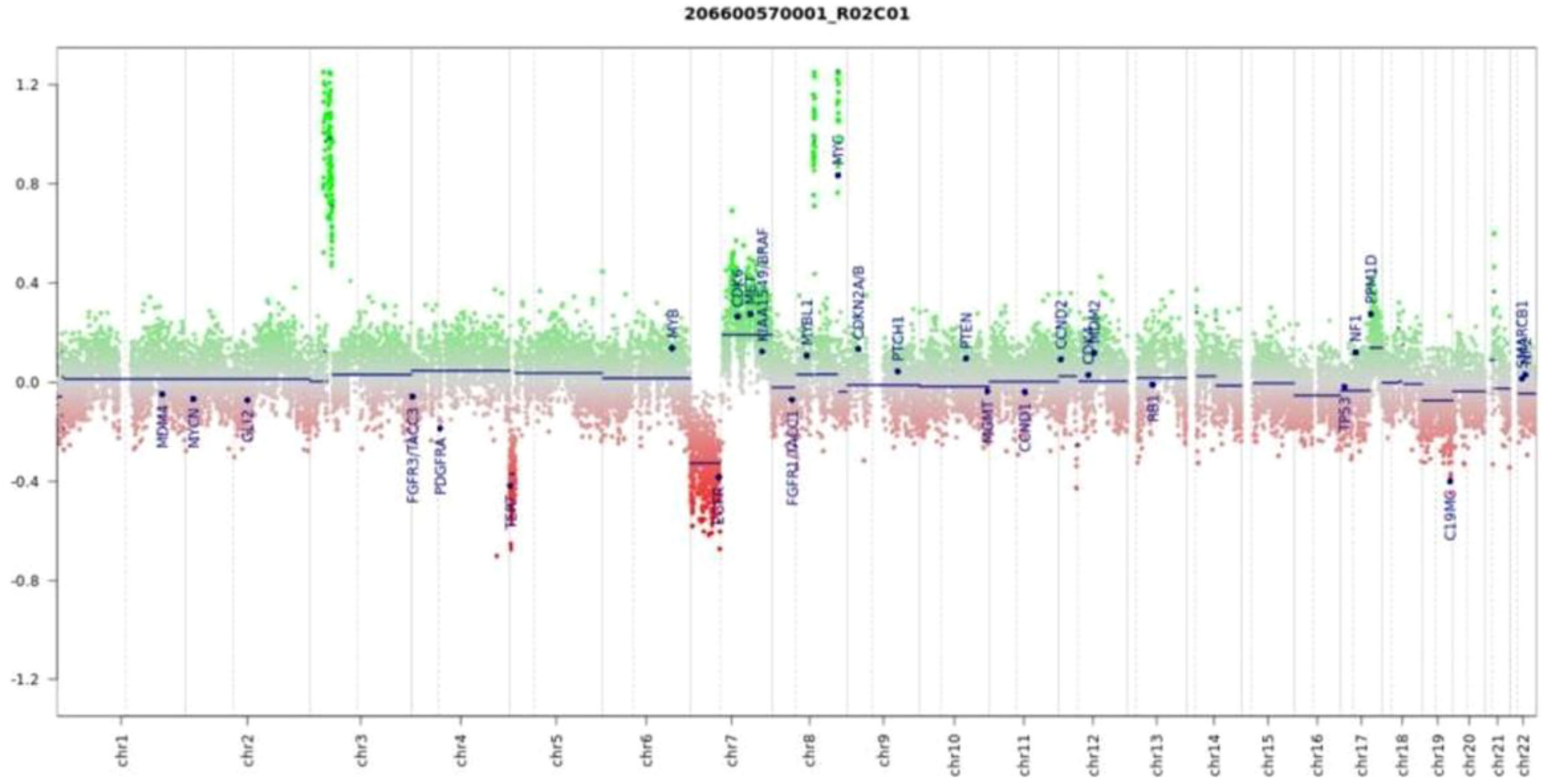
Copy number variation (CNV) analysis was performed using DNA methylation data from the Illumina EPIC 850k array. Genomic gains are visualized as upward shifts from the baseline (green), while losses appear as downward deviations (red). For easier interpretation, 29 gene regions known to be relevant in central nervous system tumors are specifically annotated (modified from the conumee package by Hovestadt & Zapatka, Bioconductor). Importantly, the profile reveals a MYC gene amplification and a deletion involving the TERT locus, both of which are alterations frequently linked to high-risk medulloblastoma.

Magnetic resonance imaging (MRI) revealed a right trigeminal nerve lesion extending to Meckel’s cave, cavernous sinus, V cranial nerve intracisternal tract, dura mater of middle cranial fossa floor near foramen ovale. Additional smaller leptomeningeal pseudo-nodular lesions were localized i) in the right postero-lateral IV ventricle, ii) among the cerebellar folia, iii) along the anterior and posterior surface of thoracic cord (**Figure 1**). Considering the possibility that the lesion was not a primary CNS tumor, a total body computed axial tomography (CT) scan was performed, which was negative. An excisional biopsy of the meningeal pseudo-nodular lesion of the IV ventricle was performed, and histological examination showed an embryonal tumor with a solid growth pattern, consisting of small cells, with high nucleus- to-cytoplasm ratio, hyperchromatic and slightly pleomorphic nuclei, sometimes arranged at the periphery of the cell, and inconspicuous nucleoli. Mitoses were brisk (50–60 per 10 HPF), and the Ki67 proliferation index was high (about 50%). The tumour expressed synaptophysin whereas YAP1 and GAB1 were negative; the expression of beta-catenin was limited to the cytoplasm. These features were suggestive of MB nonWNT/nonSHH, and methylation analysis led to the diagnosis of MB subgroup 3, subtype II, MYC amplified. NGS analysis did not identify any gene fusion, while showed VUS in PIK3CG(p.M628V), NRG1 (p.R4P), RANBP2 (p.D716N) (**Figures 2**–**4**). Tumor mutational burden (TMB) was zero.

Lumbar cerebrospinal fluid (CSF) cytology, performed 2 weeks after surgery, was positive for MB cells. Additionally, a blood test for cancer predisposition syndromes was performed using the Twist Custom Panel. Genetic testing identified the presence of a heterozygous germinal intronic variant of uncertain significance (c.-4C>T) in *CHEK2* gene (NM_007194.3), inherited from the mother.

After the biopsy was performed, less than 2 weeks after the initial imaging, the patient underwent a new MRI that showed rapid progression of the main tumor, a holocord dissemination, and a new 11- mm lesion in the right parotid gland (**Figure 1**).

Fluorodeoxyglucose (FDG) F18 positron emission tomography (PET) scan revealed pathological uptake in the leptomeningeal region of the middle cranial fossa (SUV max 31), the cavernous sinus extending to Meckel’s cave and the V cranial nerve. Additional abnormal uptake was observed in the peri- cerebellar cerebrospinal fluid spaces (SUV max 7) of the roots of C8, L4, and S1-S2 (SUV max 15) and in the right parotid gland (SUV max 20). Based on the diagnosis of metastatic MB, he was treated with “Milan strategy” protocol [16] and received post-operative methotrexate [8 g/m(2)], etoposide [2.4 g/m(2)], cyclophosphamide [4 g/m(2)], and carboplatin (0.8 g/m(2)) in a 2-month scheme with partial disease and complete metabolic response, while CSF cytology was still positive for MB cells.

He was subsequently treated with two myeloablative courses of thiotepa (300 mg/m2/d for 3 days) followed by circulating progenitor cell (CPC) administration, achieving a further reduction of posterior fossa and spinal leptomeningeal spread and CSF cytology.

He underwent cranio-spinal radiotherapy with a maximum dose to the neuraxis, trigeminal nerve, and auriculotemporal branch of the right parotid gland of 39 Gy (1.3 Gy/fraction, 2 fractions/d) and a boost of impregnation of the posterior fossa and trigeminal parotid gland to 15 Gy (1.5 Gy/fraction, 2 fractions/d). Post-radiotherapy MRI showed a partial disease response [based on RAPNO criteria (14)] with a reduction in the number and size of supratentorial and infratentorial leptomeningeal nodules (**Figure 1**). However, CSF examination revealed the presence of neoplastic cells.

Approximately two months later, he was admitted to our department due to fever, oxygen desaturation, grade 2 fatigue, and right basal pneumonia with pleural effusion without microbiological isolation, which was treated with antibiotic therapy.

After 2 weeks he underwent a re-evaluation with TB CT, which showed massive right pleural effusion and multiple pleural and peritoneal nodules (**Figure 3**). Brain and spinal MRI showed disease progression with massive leptomeningeal involvement (**Figure 3**). Bone marrow examination demonstrated disease infiltration (around 30–35% of MB cells). Therefore, thoracentesis was carried out, and cytological examination documented malignant cells. Due to the clinical and radiological progression of the disease, cytoreductive chemotherapy with three cycles of carboplatin (160 mg/m2) and etoposide (100 mg/m2) was administered for 4 days every three weeks. In the following weeks due to worsening Performance status (PS) (Lansky scale 40%), headache, vomiting, and lethargy, we decided to perform an early disease reassessment that demonstrated a multidistrict disease progression (**Figure 3**).

The patient started anti-edema therapy with dexamethasone and mannitol, and a right thoracic port- a-cath was implanted to proceed with periodic fluid evacuations with a slight clinical improvement. To ensure better supportive care, he was transferred to our palliative care center and died after one week (15 months from diagnosis).

## 3 Discussion

MBs are embryonal tumors and represent the most common malignant CNS tumors in pediatric patients. Advances in multimodal therapies over the past two decades have increased the overall survival rates of patients with standard-risk MB to 70–80%, but unfortunately, high-risk patients (younger than 3 years of age, with subtotal tumor resection and metastatic lesions at diagnosis) still have a five-year overall survival rate of about 30% (15–17).

Recent insight in molecular profiling has shown a significant heterogeneity among MBs, identifying four distinct subgroups (wingless [WNT], sonic hedgehog [SHH], group 3, and group 4),. thus allowing a more accurate patients prognostication (15, 18). Over the years the scientific evidence showed that different precursor cell populations relying on different cell signaling pathways control the development of distinct compartments from which the various subtypes of MBs arise (19). The inability of progenitor stem cells to differentiate during the growth could be the cause of MB, indeed the cancer stem cells are likely to correspond with the MB cells of origin. WNT signaling activation in the rhombic lip progenitor cells could determine WNT-activated MB, and hyperactivation SHH signaling in the cerebellar granule neuron precursors could cause SHH MB. Recent research suggests that the unipolar brush cell progenitor in the subventricular zone of the upper rhombic lip may be the cell of origin for group 4 MB, while group 3 MB appears to arise from earlier, undifferentiated progenitor cells (19, 20). Primary extra-axial MBs are reported in the literature as even rarer entities (**Table 1**). This term indicates that the lesion originates outside the brain parenchyma (5, 36) i.e. the skull, meninges, cranial nerves, and brain appendages (49). Al-Sharydah et al. reported the largest series of cases of patients with EA-MB of the posterior fossa (twenty-seven cases). They described the main radiological and histopathological features that distinguish intra-axial from EA-MB, as well as the pathological spectrum of differential diagnoses (3). Russo et al. reported a case of primary leptomeningeal medulloblastoma (MB) presenting with seizures and severe consciousness decline. MRI showed obstructive hydrocephalus and extensive leptomeningeal enhancement without solid brain lesions. Eight other similar cases are described in the literature, including five pediatric and three adult patients, two of them with initial spinal cord involvement. All of these patients showed poor prognosis after surgical treatment (5, 21).

**Table 1:** Summarizes all reported cases of primary extra-axial medulloblastomas described in the literature, a particularly rare entity characterized by its origin outside the brain parenchyma but within the central nervous system, including the skull, meninges, cranial nerves, and related structures.

Author	Age (y)/ gender	Clinica presentation	Location of MRI lesions	Treatments	Follow-up
Ferrara et al. (21)	5/M	Intracranial hypertension	Subarachnoid spaces of the posterior fossa	Surgery	Died 1 week after surgery
Suman et al. (22)	10/F	Intracranial hypertension	Subarachnoid spaces of the posterior fossa	Surgery	Died 2 weeks after surgery
Mehta et al (23)	8/M	Intracranial hypertension, dysdiadochokinesi s, left-sided dysmetria	Leptomeningeal cerebellar folia and the bilateral temporal and occipital lobes	Surgery	Died 2 months after surgery
Rushing et al (24)	30/M	Neck pain, decreased level of consciousness	Subarachnoid spaces of the posterior fossa	No treatment	Died soon after admission
Guo et al (25)	21/M	Intracranial hypertension, diplopia, tinnitus, altered state of consciousness	Leptomeningeal posterior fossa	Surgery, RT	Died 6 months after surgery
Asadollahi et al (26)	22/M	Headache, blurred vision	Dural cerebellar folia and cerebral convexity	Biopsy	Died 2 weeks after surgery
Kajtazi et al (27)	18/F	Intracranial hypertension, right leg weakness, blurred vision	Leptomeningeal sylvian fissure, posterior fossa, T6- T7	Surgery, RT	alive at 3 years follow-up
Russo et al (5)	1/M	Seizures and decreased level of consciousness	Leptomeningeal	Surgery	Died 2 months after surgery
			posterior fossa, spinal cord		
Ala et al. (28)	34/M	Headache, diplopia	Leptomeningeal	Surgery	Died 6 weeks after surgery
			Optic disc, posterior fossa, and cauda equina		
Becker RL et al. (29)	32/f	Hearing loss, ataxia, diplopia, nausea	Cerebellopontine angle	NA	NA
	52/F	Headache, vomiting,	Cerebellopontine angle	NA	NA
Akay KM et al. (30)	21/M	Headache, nausea/ vomiting, ataxia	Left posterior petrosal dura and tentorium	Surgery/RT/CT	Alive at 18 months after surgery
Salu Gil JG et al (31)	40/M	Headache, vomiting, hearing difficulties	Cerebellopontine angle	NA	NA
Fallah A et al (32)	47/F	Headache, vomiting	Cerebellopontine angle	Surgery/RT	NA
Furtado SV et al (33)	32/M	Headache, vomiting, gait unsteadiness	Leptomeningeal posterior petrous	Surgery/CT	NA
Singh M et al (7)	22/M	Headache, vomiting, facial weakness, papilledema, ataxia	Cerebellopontine angle	Surgery/RT/CT	Metastatic relapse after 15 months after surgery
Bahrami E et al (34)	23/M	Deafness, nausea/ vomiting, ataxia	Cerebellopontine angle	Surgery/RT	Alive at 1 year after surgery
Goudihalli SR et al (35)	50/M	Facial asymmetry, hearing loss	Cerebellopontine angle and porus acousticus	Surgery/RT	Vegetative state
Ratha V et al (4)	42/F	Headache, ataxia	Left posterior petrosal dura and tentorium	Surgery	Alive at 15 months
Pant I et al (36)	30/F	Headache, nausea/ vomiting	Cerebellopontine angle	Surgery	Alive at 24 months after surgery
Kumar et al (37)	9/M	Headache, vomiting, ataxia	Posterior fossa	Surgery/CT/RT	Died
	8/M	Headache, vomiting,	Posterior fossa	Surgery/RT	Died
	20/F	Headache, ataxia	Posterior fossa	Surgery/RT	alive
	24/M	Headache, vomiting,	Posterior fossa	Surgery/CT/RT	Died
Spina et al (38)	22/M	Headache, vomiting,	Cerebellopontine angle and internal acoustic meatus	Surgery/RT	NA
	26/F				
	26/F	Headache	Leptomeningeal posterior petrous	Surgery/RT	NA
Yamada et al (39)	19/F	Headache, vomiting, ataxia	Cerebellopontine angle	Surgery/Immun otherapy/RT	NA
Presutto et al. (40)	33/M	Headache, ataxia	Adjacent lateral cerebellar and fourth ventricle	Surgery	NA
Chung EJ, et al (41)	5/M	Ataxia	Adjacent lateral cerebellar	Surgery/RT	NA
Doan et al. (42)	29/M	Headache, vomiting	Tentorial	Surgery/CT/RT	NA
Ahn et al (43)	9m/F	vomiting	Cerebellopontine angle	Surgery/CT/RT	Died two months after stopping treatments
Naim‐ur‐Rahman et a (44)	3/M	Headache, vomiting, ataxia	Cerebellopontine angle	Surgery	NA
Park et al (45)	15/F	Headache, vomiting, ataxia	Left cerebellopontine angle and internal acoustic meatus	Surgery/CT/RT	NA
Santagata et al (46)	17/F	Headache, vomiting	Cerebellopontine angle	Surgery/CT/RT	NA
Nyanaveelan et al. (47)	17/F	Headache, vomiting	Cerebellopontine angle	Surgery/CT/RT	NA
Cugati et al (48)	4/F	Headache, vomiting	Left cerebellopontine angle and internal acoustic meatus	Surgery	NA

Although primary MB involving the trigeminal nerve has not been reported in the literature, there have been four cases of young adults with atypical teratoid/rhabdoid tumors, and embryonal tumors, originating from the trigeminal nerve (50–53).

Extra-neural metastases are rare, around 1–2% at diagnosis, but the incidence during follow-up can reach up to 5–10%. They are more typical of adult patients, and in almost all cases, associated with a worse prognosis (9, 54). The mechanisms underlying the extra-neural spread of MB are not yet fully understood. Surgical procedures such as craniotomy mechanically disrupt the blood-brain barrier, facilitating the seeding of tumor cells along the surgical path. Both hematogenous and lymphatic pathways involving cervical and retro-auricular lymph nodes can lead to tumor dissemination. In addition, specific molecular subgroups (notably Group 3 and 4) display an intrinsically higher propensity for leptomeningeal and distant spread, driven by pro-migratory signaling cues and clonal selection of highly invasive tumor cells that can colonize bone, bone marrow, soft tissues and lymph nodes even years after initial diagnosis.

Furthermore, iatrogenic dissemination through ventriculoperitoneal shunts is often considered as another possible mechanism, leading mainly to peritoneal metastases (55).

The first MB case with metastasis outside the CNS was described in 1936 (56). Since then, several single cases, or small series of MB ENM, have been reported in the literature (57–60). Significant differences in the location and timing of recurrence had been found between MB subgroups, which could have an impact on treatment strategies. Specifically, group 3 and group 4 tumors tend to recur with CNS spreads and extra-neural metastases rather than relapsing at the primary site, whereas SHH- activeted tumors predominantly recur at the local surgical site (49, 59).

Ali Mazloom et al. showed that most of ENM and extra-axial metastases occurs within the first 3 years and 5 years after diagnosis, respectively. They also analyzed potential prognostic factors following ENM, identifying adverse prognostic indicators such as lung or liver involvement and the time interval to develop ENM (49). Eberhart CG et al. showed that extracranial MB metastases had a relatively higher degree of anaplasia than general MB samples; the same phenomenon was associated with a higher degree of recurrence (61).

To our knowledge, our report represents the first case of primary extracranial metastatic MB subgroup 3 involving the trigeminal nerve, followed by rapid and extensive spread inside the CNS. The patient demonstrated a good sensitivity to chemo-radiotherapy, achieving a partial radiological response and complete metabolic response on extra-axial sites. However, only two months after treatment discontinuation, extracranial and intracranial disease progression was observed.

Despite the patient presenting with several high-risk features: most notably Group 3 (subtype II) and MYC amplification, the extra-axial location of the primary lesion, and metastases at diagnosis—his prognosis remained extremely poor, and he died only 15 months after the initial MB diagnosis despite multiple systemic and local treatments.

In addition, a heterozygous germline variant in the CHEK2 gene was found. The variant is annotated in ClinVar [ID: 128039], has a frequency of 0.000057 in the general population and has been classified as a variant of uncertain significance (VUS) (62).

The CHEK2 gene is an oncosuppressor gene, located at chromosome 22q12.1, which encodes a protein kinase and plays a key role in the DNA damage response (12, 13, 63).

CHEK2 gene pathogenic variants have been studied in two pediatric cohorts of six patients each with different tu, ours, including neuroblastomas, non-Hodgkin’s lymphomas, thyroid tumors, melanomas, sarcomas, and brain tumors. Interestingly in the latter group there were two MBs (12, 64).

However, the CHEK2 variant described in our case has so far only been considered a VUS. Functional studies are needed to clarify its role in tumor development and/or metastasis.

MBs remain a challenging and heterogeneous group of embryonal tumors, with significant variations in clinical presentation, molecular profiles, and outcomes. This report details a notably rare case of primary extra-axial MB involving the trigeminal nerve, which was distinguished by its rapid progression both within and outside the central nervous system. The exceptional rarity of such primary EA-MBs, along with their generally poor prognosis, highlights the critical need for improving diagnostic techniques, and tailoring treatment plans. Advancements in molecular profiling and treatment strategies are essential for the more effective management of this challenging disease.

## 4 Materials and methods

The patient and his legal guardians provided informed consent for the study. All clinical data were collected from the electronic medical record of the patient. A centralized review of the histological characterization was conducted.

### 4.1 Genetic testing and data analysis

Genomic DNA was extracted, using the DNA Blood Mini Kit (Qiagen, Hilden, NW, Germany) according to the manufacturer’s instructions, from circulating leukocytes of peripheral blood samples, after obtaining informed consent. DNA quantification was performed by Qubit fluorimeter (Life Technologies, Carlsbad, California, USA) with the dsDNA HS Assay kit following the manufacturer’s instructions. The genetic analysis was performed through Next Generation Sequencing (NGS) by using a custom clinical exome panel containing more than 8, 500 genes, including those involved in DNA cancer-predisposition syndromes (Twist Bioscience, South San Francisco, CA, USA) on a NovaSeq 6000 platform (Illumina, San Diego, CA, USA).

The genes analyzed by using a custom clinical exome panel were: APC (NM_000038.6), BRCA1 (NM_007294.4), BRCA2 (NM_000059.3), CHEK2 (NM_007194.4), DICER1 (NM_030621.4), MLH1 (NM_000249.4), MSH2 (NM_000251.3), MSH3 (NM_002439.5), MSH6 (NM_000179.2), PALB2 (NM_024675.4), NBN (NM_002485.5), PMS2 (NM_000535.5), PTCH2 (NM_003738.5), SMARCA4 (NM_003072.4), TP53 (NM_000546.6), NF2 (NM_000268), LZTR1 (NM_006767.4), POLE (NM_006231.4), MUTYH (NM_012222.2), CREBBP, ELP1, EP300, GPR161, NTHLI, PTCH1, PTPN11, SUFU, SMARCB1.

### 4.2 NGS analysis in tumor tissue

DNA was extracted from formalin-fixed paraffin-embedded tumor tissue using the Maxwell CSC instrument (Promega, Madison, USA) with the Maxwell RSC DNA FFPE kit (Promega, Madison, USA) according to the manufacturer’s protocol; DNA concentrations were measured on a Qubit2.0 Fluorometer (Thermofisher Scientific, Waltham, USA) using the Qubit dsDNA High Sensitivity. A comprehensive genomic profiling assay was performed in NGS and targeting 523 cancer-relevant genes. NGS data were analyzed with Illumina TruSight Oncology 500 Local App v2.1, and variant report files were uploaded into the Pierian Clinical Genomics Workspace cloud (Pierian DX software CGW_V6.21.1).

### 4.3 Methylation profiling and molecular classification

Genome-wide DNA methylation profiling was performed using the Illumina Infinium MethylationEPIC BeadChip array (850k). The raw data (IDAT files) were analyzed using the brain tumor classifier version 12.5 developed by the German Cancer Research Center (DKFZ, Heidelberg, Germany) to assign a methylation class and subclass.

### 4.4 Literature review methodology

A comprehensive bibliographic search was conducted in the PubMed, Scopus, Web of Science, and Embase databases. The search terms used included “medulloblastoma, “ “extraneural metastases, “ “extracranial metastases, “ “trigeminal nerve, “ “extra-axial, “ “primary medulloblastoma, “ “cancer predisposition, “ and “germline pathogenic variants.” Articles describing atypical presentations of medulloblastoma with extra-axial origin or extraneuronal spread, both at diagnosis and during the course of the disease, were included. Only peer-reviewed studies published in English were considered, such as case reports, case series, and reviews relevant to the topic. The data collected were then summarized and critically discussed in relation to the clinical case presented.
