# Supplementary material for: Metastatic extra-axial medulloblastoma involving the trigeminal nerve: a rare prognostic entity with a comprehensive literature review
Source: Front Oncol. 2026 Jan 19;15:1573781. doi: 10.3389/fonc.2025.1573781 (PMC12861904; doi:10.3389/fonc.2025.1573781)
Supplement: Supplementary file 1 [file Presentation1.pptx]

## Slide 1
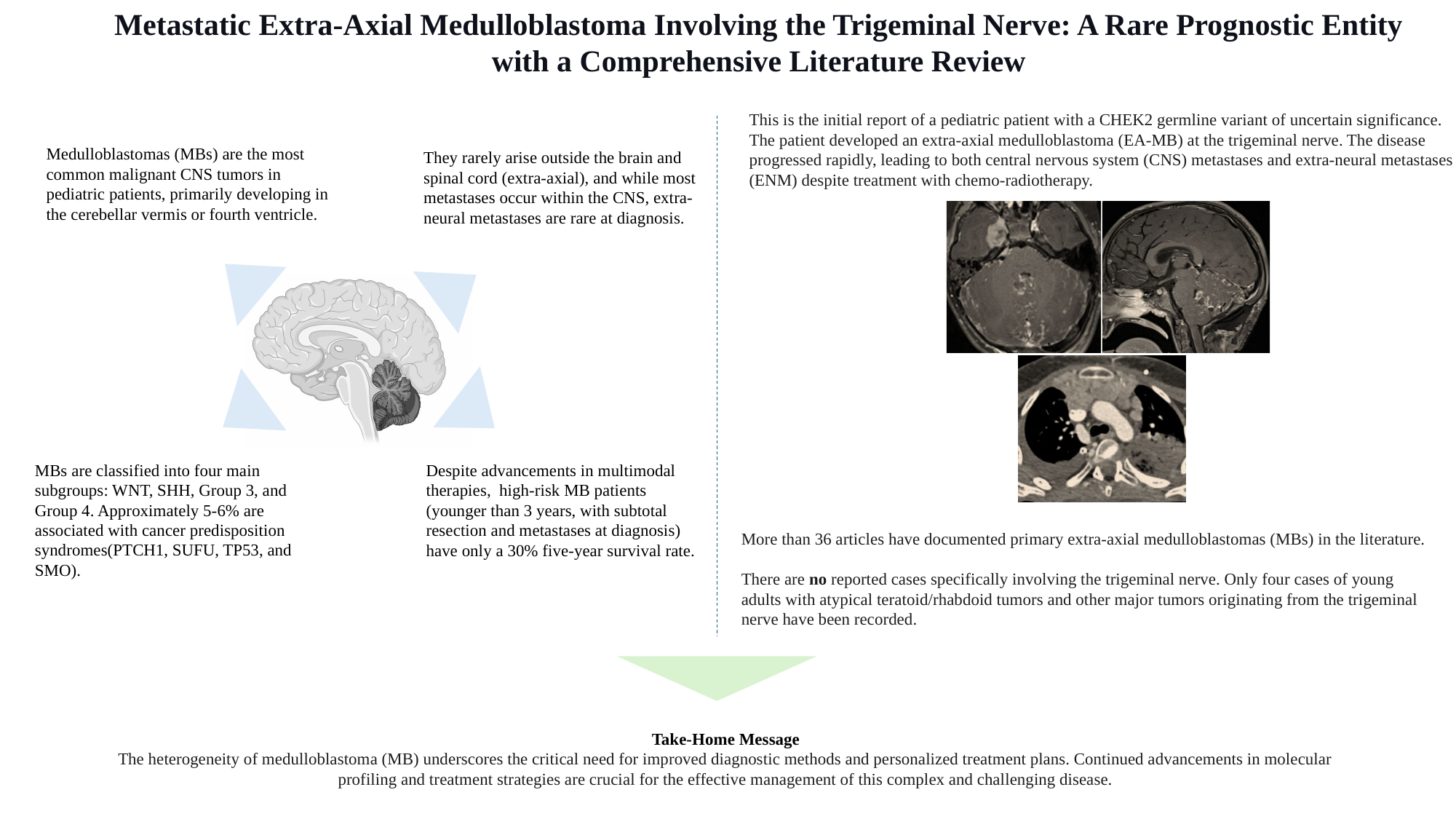

Metastatic Extra-Axial Medulloblastoma Involving the Trigeminal Nerve: A Rare Prognostic Entity with a Comprehensive Literature Review
This is the initial report of a pediatric patient with a CHEK2 germline variant of uncertain significance. The patient developed an extra-axial medulloblastoma (EA-MB) at the trigeminal nerve. The disease progressed rapidly, leading to both central nervous system (CNS) metastases and extra-neural metastases (ENM) despite treatment with chemo-radiotherapy.
Medulloblastomas (MBs) are the most common malignant CNS tumors in pediatric patients, primarily developing in the cerebellar vermis or fourth ventricle.
They rarely arise outside the brain and spinal cord (extra-axial), and while most metastases occur within the CNS, extra-neural metastases are rare at diagnosis.
MBs are classified into four main subgroups: WNT, SHH, Group 3, and Group 4. Approximately 5-6% are associated with cancer predisposition syndromes(PTCH1, SUFU, TP53, and SMO).
Despite advancements in multimodal therapies, high-risk MB patients (younger than 3 years, with subtotal resection and metastases at diagnosis) have only a 30% five-year survival rate.
More than 36 articles have documented primary extra-axial medulloblastomas (MBs) in the literature.
There are no reported cases specifically involving the trigeminal nerve. Only four cases of young adults with atypical teratoid/rhabdoid tumors and other major tumors originating from the trigeminal nerve have been recorded.
Take-Home Message
The heterogeneity of medulloblastoma (MB) underscores the critical need for improved diagnostic methods and personalized treatment plans. Continued advancements in molecular profiling and treatment strategies are crucial for the effective management of this complex and challenging disease.
